# Reviewer Acknowledgments

**DOI:** 10.1002/kjm2.70306

**Published:** 2026-10-15

Consistent high quality of papers published in “Kaohsiung Journal of Medical Sciences” can only be maintained with the cooperation and dedication of a number of expert referees. The Editors would like to thank all those who have donated the hours necessary to review, evaluate, and comment on manuscripts; their conscientious efforts have enabled the journal to maintain its tradition of excellence. We are grateful to the following reviewers for their contributions during 2026.

**B**

Bair Ming‐Jong

**C**

Chang Hong‐Yi

Chang Hsueh‐Wei

Chang Jung‐Shan

Chang Kee‐Lung

Chang Long‐Sen

Chang Shih‐Ching

Chang Ting‐Ting

Chang Wei‐Chiao

Chang Wei‐Hsiang

Chang Wen‐Tsan

Chang Yang‐Pei

Chang Yo‐Chen

Chao Pin‐Zhir

Chen Cheng‐Sheng

Chen Chia‐Hsiang

Chen Chia‐Hsin

Chen Chien‐Hung

Chen Chien‐Lin

Chen Chi‐Hau

Chen Chih‐Hwa

Chen Fang‐Ming

Chen Hsin‐Yi

Chen I‐Chen

Chen Jen‐Ruei

Chen Ming‐Jen

Chen Po‐Jen

Chen Szu‐Chia

Chen Wen‐Pin

Chen Ya‐Fang

Chen Yong‐Chen

Chen Yung‐Chia

Cheng Hung‐Chi

Cheng Kuang‐Hung

Cheng Meng‐Hsuan

Cheng Pin‐Nan

Cheng Tsung‐Lin

Chiang Bor‐Luen

Chiang Chih‐Kang

Chien Chih‐Yen

Chiu Hung‐Wen

Chiu Ing‐Ming

Chiu Yen‐Cheng

Cho Shih‐Feng

Chou Chung‐Hsing

Chou Shah‐Hwa

**D**

Ding Dah‐Ching

**F**

Fang I‐Mo

Feng I‐Jung

**G**

Gau Yuh‐Ching

Ge Yan‐Zhi

**H**

Hsiao George

Hsieh Ming‐Hsiung

Hsieh Yih‐Hsien

Hsu Chih‐Yao

Hsu Chin‐Hsin

Hsu Chung‐Yao

Hsu Jia‐Wei

Hsu Li‐Sung

Hsu Po‐Chao

Hsu Sheng‐Min

Hsu Shih‐Ping

Hsu Yao‐Chun

Hsueh Yuan‐Shuo

Huang A‐Mei

Huang Bin

Huang Chun‐Ming

Huang Hung‐Ling

Huang Jiun‐Chi

Huang Pei‐Ming

Huang Peng

Huang Shu‐Chien

Huang Shu‐Pin

Huang Tze‐Sing

Huang Wei‐Chien

Huang Wen‐Chung

Huang Yi‐Shuian

Hung Chao‐Hung

Hung Chih‐Hsing

Hung Jan‐Jong

Hung Shih‐Ya

Hung Szu‐Chun

Hung Wen‐Chun

**J**

Jhuo Shih‐Jie

Jou Jwo‐Huei

**K**

Kang Lin

Kao Shao‐Hsuan

Kao Wei‐Yu

Ku Hui‐Chun

Kuo Cheng‐Hsiang

Kuo Chin‐Sung

Kuo Hann‐Chorng

Kuo Li‐Jen

Kuo Ting‐Chun

Kuo Wen‐Huang

**L**

Lai Chun‐Liang

Lan Sheng‐Hui

Lee Chien‐Hsing

Lee Chyi‐Long

Lee Hsiang‐Chun

Lee I‐Cheng

Lee Kuei‐Chuan

Lee Kuen‐Haur

Lee Kun‐Ze

Lee Tsong‐Hai

Lee Yen‐Mei

Lee Yuan‐Chii

Lei Wei‐Yi

Li Chia‐Yang

Li Hung‐Yuan

Li Jun‐Jie

Liang Chia‐Hua

Liang Jin‐Tung

Lin Chi‐Chien

Lin Chien‐Chung

Lin Chun‐Hung

Lin Fan‐Li

Lin Hui‐Ju

Lin Ming‐Hong

Lin Peng‐Chan

Lin Ping‐Ting

Lin Yih‐Jyh

Lin Yi‐Hsiung

Lin You‐Hsien

Liu Ping‐Yen

Liu Yen‐Chin

Liu Yi‐Chang

Liu Yu‐Li

Loh Joon‐Khim

Long Cheng‐Yu

Lu Ming‐Chi

**M**

Mao En‐Qiang

**P**

Pan Hung‐Chuan

Peng Cheng‐Yuan

Peng Chung‐Kan

Peng Yen‐Ping

**S**

Shen Ming‐Yi

Shiah Shine‐Gwo

Su Pei‐Yi

Su Tung‐Hung

Suen Jau‐Ling

Sun Jui‐Sheng

Sung Jin Yong

**T**

Tai Ta‐Wei

Tang Chih‐Hsin

Tang Gau‐Jun

Ting Chien‐Kun

Tsai Chia‐Kuang

Tsai Kuo‐Wang

Tsai Wei‐Chung

Tsai Yi‐Chun

Tseng Cheng‐Hao

Tu Hung‐Pin

Tyan Yu‐Chang

**W**

Wang Cheng‐Ping

Wang Chih‐Wen

Wang Chih‐Yuan

Wang Jieh‐Neng

Wang Jing‐Houng

Wang Yan‐Hsiung

Wang Yen‐Yun

Wang Yi‐Ching

Wang Yuan‐Hung

Wei Chun‐Wang

Wei Wei‐Dong

Weng Jie

Wu Chin‐Chen

Wu Chin‐Chung

Wu Chun‐Chi

Wu Horng‐Jiun

Wu Hung‐Tsung

Wu I‐Chen

Wu Jeng‐Yih

Wu Kun‐Pin

Wu Ming‐Heng

Wu Ping‐Hsun

Wu Shang‐Ju

Wu Shang‐Yin

Wu Shan‐Ying

Wu Sheng‐Wen

Wu Shyh‐Jong

Wu Zhi‐Fu

**Y**

Yang Jai‐Sing

Yang Pei‐Ming

Yang San‐Nan

Yang Shun‐Fa

Yang Yu‐Chiao

Yeh Jwu‐Lai

Yeh Kun‐Yun

Yeh Ming‐Lun

Yeh Shu‐Lan

Yoshinaga Jun

Yu Dah‐Shyong

**Z**

Zou Yu‐Tian

